# Effects of Dominant/Subordinate Social Status on Formalin-Induced Pain and Changes in Serum Proinflammatory Cytokine Concentrations in Mice

**DOI:** 10.1371/journal.pone.0080650

**Published:** 2013-11-20

**Authors:** Marjan Aghajani, Mohammad Reza Vaez Mahdavi, Mohsen Khalili Najafabadi, Tooba Ghazanfari, Armin Azimi, Saeid Arbab Soleymani, Shirin Mahdi Dust

**Affiliations:** 1 Department of Physiology, Faculty of Medical Sciences, Shahed University, Tehran, Iran; 2 Equity and Health research Department, Shahed University, Tehran, Iran; 3 Department of Immunology, Faculty of Medical Sciences, Shahed University, Tehran, Iran; 4 Traditional Medicine Clinical Trial Research Center, Shahed University, Tehran, Iran; 5 Department of Physiology, Faculty of Medical Sciences, Tehran Medical University, Tehran, Iran; University of Sao Paulo, Brazil

## Abstract

Current investigations regarding social stress primarily focus on the health consequences of being in stressful social hierarchies. The repetitive nature of social conflicts seems to favor an induction of hyperalgesia or hypoalgesia, both in rodents and humans. Additionally, social conflicts may affect the immune system. In order to better establish the pain and immune responses to stress, the present study implemented a sensory contact model on 32 male BALB/c mice. Subsequent to establishing a dominance/submissive social relationship, each mouse was injected with formalin (20 μl, 2%) and their pain behavior was scored and serum concentrations of proinflammatory cytokines IL-1 and IL-6, and corticosterone were also measured. Test results revealed that subordinate mice were hypoalgesic during chronic phase of formalin test compared to control and dominant mice (P<0.05). On the other hand, subordinate mice were hyperalgesic compared to dominant mice during the whole acute phase of formalin test (P<0.05). Corticosterone, IL-1 and IL-6 concentrations were much higher in serum of dominant and subordinate mice than in the control group (p<0.05). The results indicated that, although both dominant and subordinate animals displayed an increase in serum corticosterone and proinflammatory cytokines during social interactions, their response to pain perception differently was affected with the social status.

##  Introduction

Current literature regarding social stress primarily indicates negative health consequences of being in a stressful social hierarchy. A person’s social ranking has a huge effect on his/her level of stress [1,2]. Although stress is not a disease unto itself, continuous exposure to stressful stimuli has been directly related to onset, progression or outcome of pathological processes [3,4]. Sapolsky et al found that there are lower levels of stress hormones in high-ranking baboons compared to submissive baboons while subordinate animals displayed increased heart rates and higher blood pressure, which have a negative impact on health [5].

A wide variety of experimental and clinical investigations have shown that both natural and laboratory-induced stressors have a profound influence on immune response [6-8]. Chronic stressors can have health-adverse effects, some of which are mediated through immune mechanisms [9]. Additionally, acute stress-induced immunoenhancement may serve to increase immunoprotection. In contrast, stress may exacerbate immunopathology, if the enhanced immune response is dysregulated following prolonged activation, as seen during chronic stress [9]. Chronic stress has also been shown to alter the expression of cytokines, such as interleukin (IL)-1 and IL-6 [10]. In studies of the splenocytes of subordinate animals, high levels of the proinflammatory cytokines including IL-6 and tumor necrosis factor (TNF)-α were secreted [11-13]. Stressful experiences can also directly provoke transient increases in proinflammatory cytokines of plasma and brain, especially IL-1 and IL-6 [14].

Laboratory and clinical studies have also revealed that social interactions between pairs of conspecifics can affect the response of individuals to external stimuli [15]. There has been growing interest in the relationship between pain and psychiatric pathologies in the past 20 years, with a focus on sensory mechanisms of nociception in animals [16]. Studies of chronic stress suggest that stress can produce hyperalgesia rather than hypoalgesia [16,17]. Alternatively, it has been shown that, in animal models of pain, exposure to a new circumstance, or a potentially fear-inducing or stressful situation, actually reduces pain reactivity [18,19].

The social subordinate/dominant paradigm represents a psychological chronic stress protocol, which lends itself to a natural experiment of the physiological alterations induced by the chronic stress stimulus [3,20]. Group-housed male mice establish social hierarchies, the loss of hierarchical position in a group or the territory appears to be a key factor in determining the occurrence of chronic state of stress [21]. Utilizing these aforementioned models, the first objective of the current study was to examine the relationship between dominant/subordinate social status and the perception of pain. For this purpose, prolonged chemically evoked pain behavior was assessed in the formalin model. The second aim of this study sought to establish an immune response to social stress. We measured serum corticosterone, IL-1 and IL-6 concentrations, with the hypothesis that both dominant and subordinate males would show an increase in serum inflammatory cytokines and corticosterone concentrations in response to the stress of social interaction.

##  Experimental Procedures

### Animal Model and Experimental Protocol

The experimental subjects were 32 naive, adult male Balb/c mice (aged 8–10 weeks). The mice, were born, reared and housed in same-sex sibling groups (four to eight per cage) in Plexiglas cages at the Pasteur Institute (Karaj, Iran). When the subjects became adults (aged 8–10 weeks), they were obtained from Pasteur Institute. Ambient temperature was maintained between 22 and 24 °C, and the vivarium was maintained under a 12:12 h light/dark cycle. Food and water were available ad libitum. On arrival, all subjects were weighed and assigned into two groups: 1) control group (n=8); 2) stress group (n=24). Control animals remained group-housed [22] while stress mice were housed in cages which were divided in two parts by a wire-mesh partition and one mouse was placed in each of the two compartments (so there were 2 male mice per cage in stress group and both animals had access to water and food ad libitum and independently of each other). In this housing arrangement, animals were prevented from fighting, but they could see, hear, and smell each other. This trans-grid sensory contact is regarded as passive social contact and may alleviate symptoms of isolation. However, housing mature male mice with sensory contact through a grid divider demonstrated no beneficial effect, but instead led to distress and potential impairment of their well-being [23]. This model of social stress sensory contact has been reported to prolong the stress effects of defeat in the subordinate animals [24]. 

Although some studies revealed that mice of the Balb/c inbred strain show relatively low levels of social interaction [25-28], other investigations indicated that males of this strain show inter-strain aggression and form dominant/subordinate relationships reliably when housed together in cages [29,30]. After two weeks of habituation period the partition was removed for 10 minutes daily (for a total of 6 days) in stress group, and the two animals were allowed to interact freely and attack each other. If the interaction provoked wounds in mice, it was interrupted by lowering the partition. The dyad was considered stable when one of the two mice achieved the dominant social rank (i.e., for 3 consecutive days the dominant and subordinate roles did not change) [15]. Systematic daily observations and evaluations yielded 9 dyads which developed clear and stable dominant-subordinate roles for the constituent individuals. Three dyads were excluded from the study because they could not develop a dominant/subordinate relationship by the end of the week of agonistic encounters.

Twenty four hours after termination of the last direct social interaction (i.e. at day 7 after starting the stress protocol), the animals were weighed again and underwent a standard test of pain responses to subcutaneous formalin injection.

The original research reported herein (involving animals and their care, experimental protocols and procedures) were approved by Institutional Animal Care and Use Committee (IACUC) at the Medical University of Shahed (Iran). This study was also conducted in accordance with the Guide for the Care and Use of Laboratory Animals as adopted and promulgated by the European Communities Council Directive of 24 November 1986 (86/609/ EEC). Additionally, all efforts were made to minimize animal suffering and to use only the number of animals necessary to produce reliable scientific data [31]. Twenty four hours after formalin test, mice were anesthetized slightly with ether. Blood samples were obtained from the heart ventricle [32] and the mice were subsequently sacrificed in ether chamber. To measure proinflammatory cytokines and corticosterone concentrations, blood samples were collected in tubes and allowed to clot on ice. Then, the samples were centrifuged immediately at 3000 rpm for 10 minutes and serums were separated and stored at -70°C. 

## Nociceptive Assay

Experiments took place between 12:00 and 16:00. Briefly, 20 μl of 2% formalin (a relatively mild concentration - 1:50 dilution of 37% formalin solution in double deionized H2O) was injected into the plantar surface of the right hind-paw. Mice were standing on a glass floor within Plexiglas observation cylinders (30 cm diameter; 25 cm high), and were habituated to these cylinders for 30 minutes before the formalin injection. The mice were removed, injected, and replaced in the cylinder [15] and their pain response was scored every 15 seconds for 60 minutes according to the scale listed below:

1 ***Nopain***: Normal weight born on injected paw
2 ***Favoring***: Injected paw in contact with the floor, but full weight not on the paw
3 ***Lifting***: Injected paw elevated
4 ***Licking***: Licking or biting the injected paw

The subcutaneous injection of diluted formalin produces a biphasic nocifensive behavioral response in mice. The early phase consists of intense licking and biting of the injected paw during the first 6 minute interval, and reflects the behavioral response to acute pain (phase I), while the second phase of licking and biting occurs 15 to 35 minutes after formalin injection and represents the behavioral response to chronic pain (phase II). In between the two phases, there is an inter-phase in which pain behavior (paw-licking and lifting) is almost reduced to zero [33]. Behaviors were measured by two experienced observers who were blind to the implemented conditions.

### Corticosterone Assay

A commercial serum corticosterone radioimmunoassay kit (ADI-900-097; Enzo Life Sciences) was used to evaluate serum concentration of corticosterone in all mice. The assay had high and low limits of detectability of 5 and 1000 ng/mL, respectively according to a standard curve. All procedures were performed according to the manufacturer's instructions.

### Immunological Assay

Circulating immunoreactive IL-6 and IL-1β levels were measured using commercially available quantitative enzyme-linked immunosorbent assays (R&D Systems Europe, Abingdon, UK) [34]. The assays did not measure biological activity of the cytokines. Standard sensivity assays were used and the manufacturers reported the sensivity thresholds in serum as 0.7 pg/ml and 1.5 pg/ml for IL-6 and IL-1β, respectively. All measurements were made by a single trained individual to avoid interobserver variation.

### Statistical Analysis

Statistical analyses were performed using the Sigma Stat software (SystatSofware, Inc., Point Richmond, CA, USA). Analyses included two-way ANOVA test (the relation of dominant/subordinate social status with the mean nociceptive score in acute phase, interphase and chronic phase of the formalin test) and one-way ANOVA test (statistical significance for cytokines and corticosterone concentrations) followed by the post hoc Dunnett’s test and the post hoc Bonferroni’s test for multiple comparisons. Additionally, the results of pain behaviors after formalin injection were analyzed with a one-way ANOVA for repeated measures (10 blocks) to test the difference between all blocks in each group. A significance level of p<0.05 was used in all cases. Data are presented in the text and in all figures as means ± SEM.

## Results

### Body Weight

**Figure 1:**
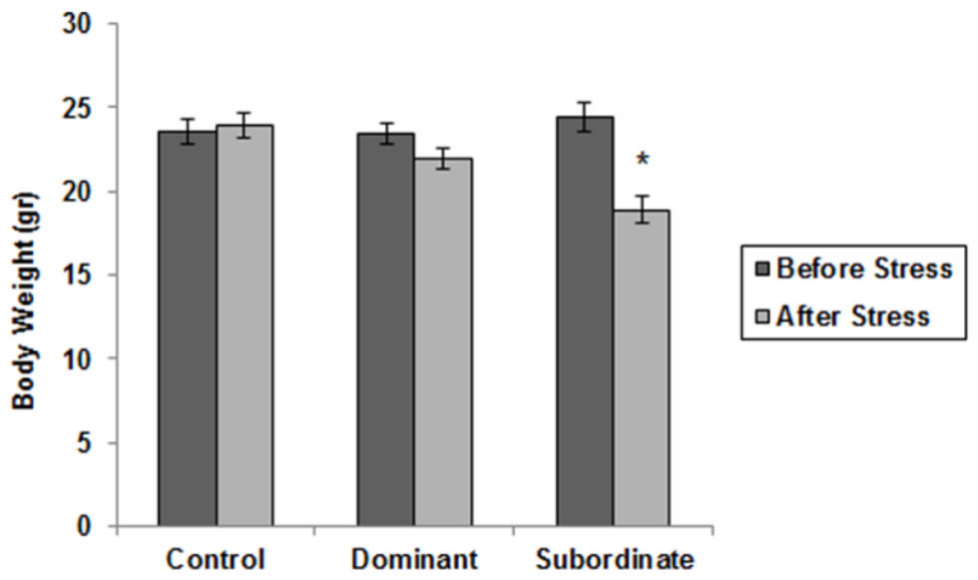
Body weight changes in mice exposed to chronic social stress. Experience of subordinate social status caused significant decrease in their body weight. *p < 0.05: beginning of study vs. end of chronic social stress (Data are means ± SEM; Controls: n=8, Dominants and subordinates: n=9).

Control and all experimental animals were similar in body weight before the procedure. As shown in Figure **1**, body weight of subordinate subjects was less than their initial body weight (p<0.05). Dominant social status resulted in weight loss but this decline was not considerable (p=0.081). This finding served to indicate that subordinate social paradigm has caused noticeable weight loss.

### Comparison of Pain Perception between Control and Dominant/Subordinate Groups

**Figure 2:**
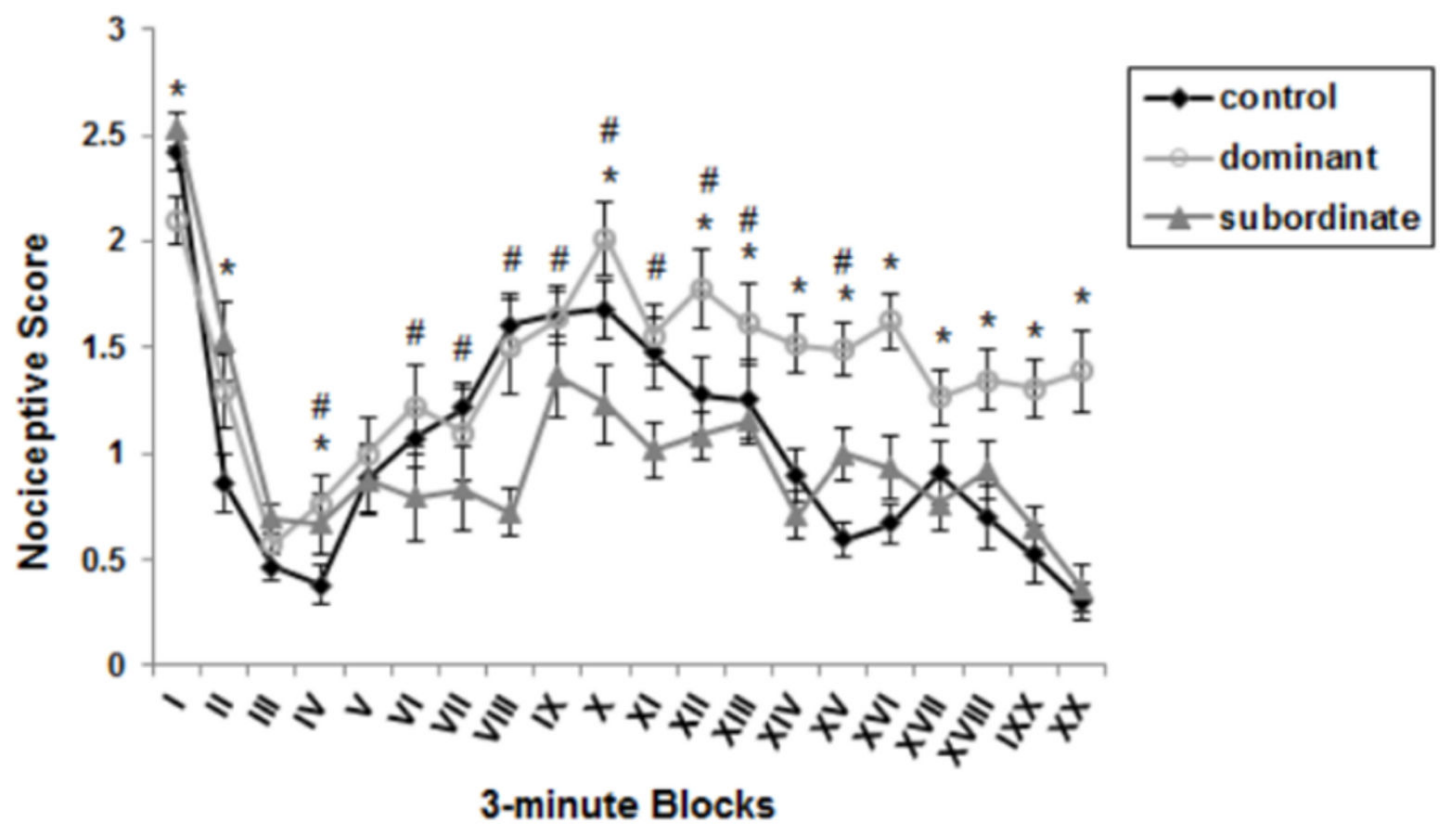
Effect of social dominant/subordinate social status in the model of formalin-induced pain Pain scores as a function of time. The observation period is divided into twenty 3-minute blocks (the average scale of each minute for each groups was calculated, and the mean of each 3 minutes was regarded as one block). Data are means ± SEM (Controls: n=8, Dominants and subordinates: n=9). *p < 0.05: significant difference between dominant and control groups in 3-minute Blocks. # p < 0.05: significant difference between subordinate and control groups in 3-minute Blocks.

An one-way ANOVA for repeated measures revealed the characteristic biphasic curve of the formalin-induced behavioral response in control group. The first peak of licking behavior during the first 6 minute block reflects the behavioral response to acute pain, whereas the second part of this curve from minute 15 to minute 35 after formalin injection represents behavioral response to chronic pain. In between these two phases, there was an inter-phase (from minutes 7 to 14) in which licking behavior was almost reduced to zero [15,33]. The observational period was divided into twenty 3-minute blocks (the average scale of each minute for each groups was calculated, and the mean of 3 minutes was regarded as one block). A repeated measure one-way ANOVA was used to compare 3-minute blocks with each other in a group and this analysis revealed statistical difference between these blocks during formalin test. As shown in Figure **2**, a significant difference (p<0.05) was observed between control and dominant mice in all blocks except for blocks 3, 5, 6, 7, 8, 9 and 11. Moreover, significant differences (p<0.05) between subordinate and control groups were observed in blocks of 2, 3, 4, 6, 7, 8, 9, 10, 11, 12, 13 and 15 (Figure **2**).

**Figure 3:**
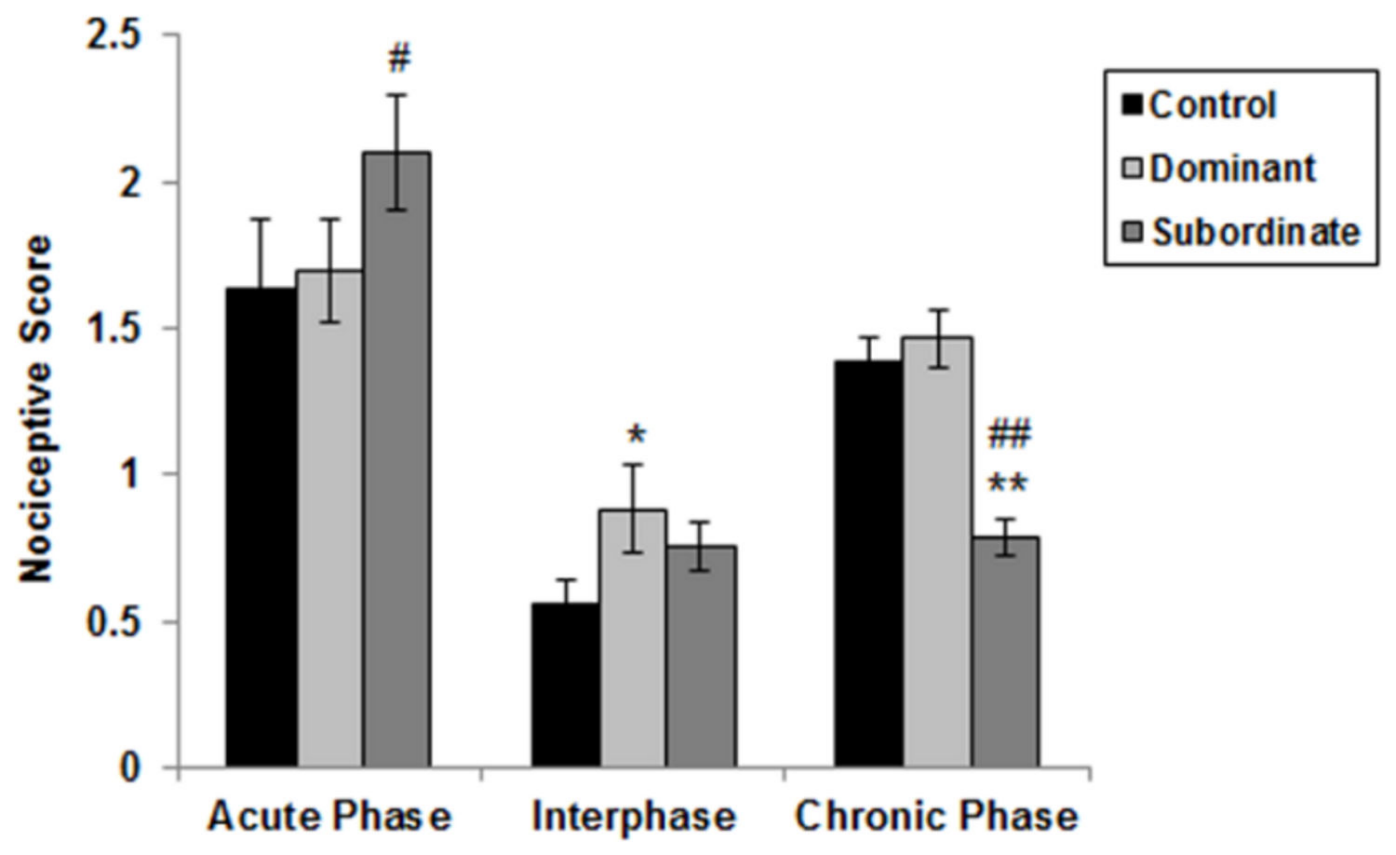
Effect of social dominant/subordinate social status in the model of formalin-induced pain Cumulated pain scores during the acute phase (cumulated pain scores from 0 to 6 min), interphase (cumulated pain scores from 7 to 14 min), and late phase (cumulated pain scores from 15 to 60 min). Symbols/bars represent mean ± SEM pain scores per group. (Controls: n=8, Dominants and subordinates: n=9). * p < 0.05, ** p < 0.001:: significant difference between dominant/subordinate animals and controls. ^#^ p < 0.05, ^##^ p < 0.001: significant difference between dominant and subordinate animals.

Mean nociceptive scores of each group were reported for each phase of formalin test (Figure **3**). The average scale of each minute for each group was calculated using data presented in Figure **2**. Subsequently, the mean and SEM of these average numbers from minute 1 to 6 was considered as acute phase, from minute 7 to 14 as interphase, and from minute 15 to 35 as chronic phase. As shown in Figure **3**, chronic pain perception was affected by subordination, but dominant status in a dyad caused no difference between control and dominant mice. Specifically, Figure **3** reveals that implementing such stressor caused significant decrease of chronic pain sensation (hypoalgesia) in formalin test of subordinate mice compared to control group (control: 1.389±0.0822 vs. subordinate: 0.785±0.0633; p<0.001). No statistical difference was observed between the dominant and the control groups in the chronic phase of the formalin test (control: 1.389±0.0822 vs. dominant: 1.464±0.0988; p=0.556). Due to observed significant differences in the final blocks between the dominant and control groups, we compared pain response of from minute 36 to 60 after formalin injection (data is not displayed in Figure **3**); this analysis revealed that pain perception in dominant mice during this time was significantly higher than controls (control: 0.768±0.057 vs. dominant: 1.455±0.0732; p<0.001). A comparison of dominant and subordinate mice in chronic phase of the formalin test revealed a significance of p<0.001, in which pain perception of dominants was higher as compared to subordinate mice. Moreover, this figure shows no difference in the acute phase of the formalin test between control and both dominant (control: 1.636±0.236 vs. dominant: 1.694±0.174; p=0.979) and subordinate groups (subordinate: 2.069±0.186 vs. control: 1.636±0.236; p=0.181). Significant difference was found, however, between the subordinate and the dominant mice in acute pain sensation after formalin injection (p=0.028) with the subordinate animals displaying higher pain perception. A repeated measure one-way ANOVA to compare pain phases with each other within a group; results of this statistical analysis are shown in Table **1** (these statistical differences are not shown in Figure **3**). 

### Comparison of Serum Corticosterone Level between Control and Dominant/Subordinate Groups

**Table 1:** Statistical differences between the acute, interphase, and chronic phase in experimental groups in a model of formalin test.

*Groups*	**Acute Phase vs Chronic Phase *p value***	**Interphase vs Chronic Phase *p value***	**Acute Phase vs Interphase *p value***
*Controls*	0.047	0.012	<0.001
*Dominants*	0.082	0.039	0.027
*Subordinates*	<0.001	0.154	<0.001

A significance level of p<0.05 was used in all cases.

**Figure 4:**
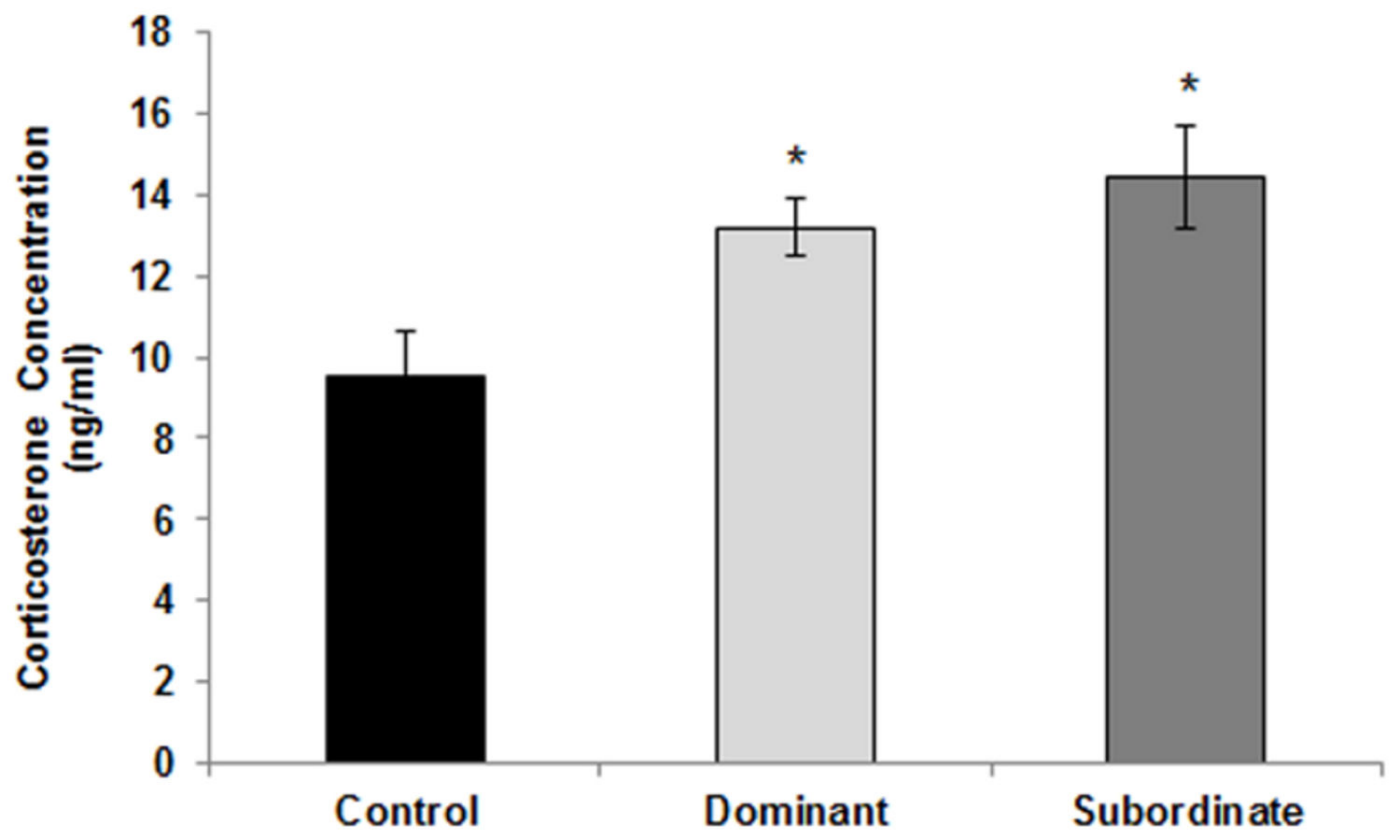
Effect of social status on serum concentration of corticosterone in mice. Data are means ± SEM (Controls: n=8, Dominants and subordinates: n=9). * p < 0.05: significant difference between dominant/subordinate animals and controls.

Evaluation of corticosterone concentration in serum of experimental subjects (Figure **4**) revealed that corticosterone level was increased significantly in both dominant (mean ± SEM: 13.201±0.732 ng/ml) and subordinate (mean ± SEM: 14.463±1.27 ng/ml) mice as compared to controls (mean ± SEM: 9.522±1.149 ng/ml; p<0.05), and there was no difference between dominant and subordinate mice (p=0.724).

### Comparison of Serum Proinflammatory Cytokines Levels between Control and Dominant/Subordinate Groups

**Figure 5:**
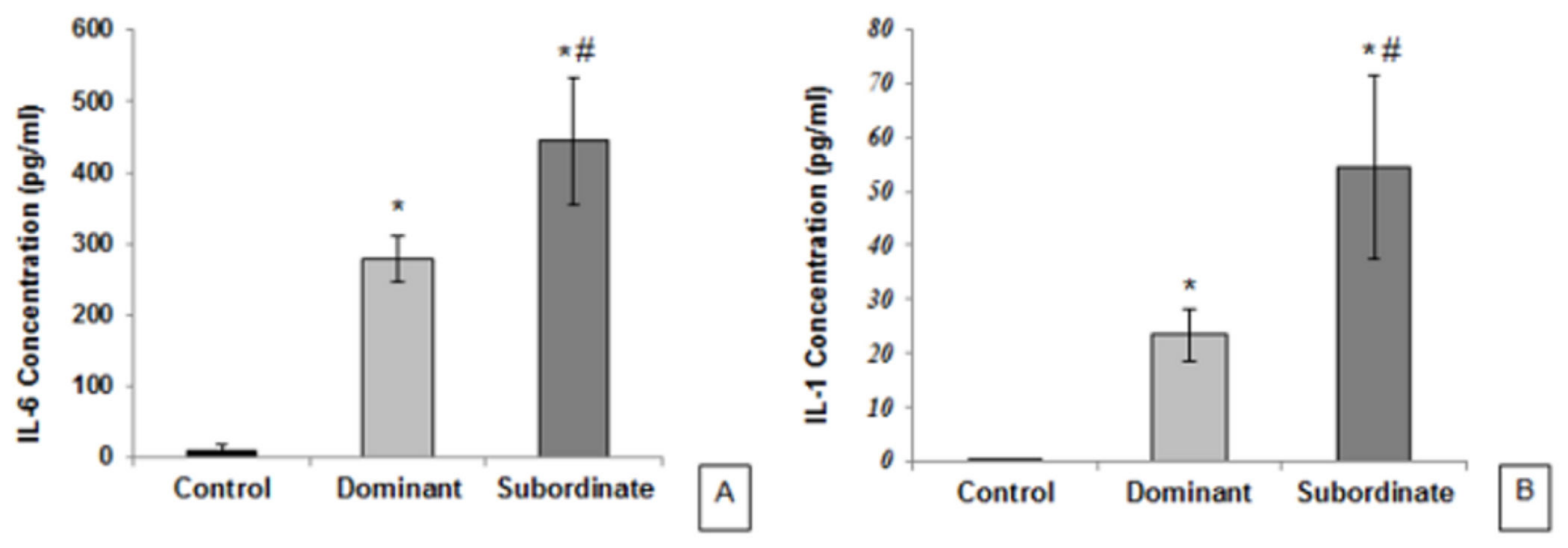
Effect of social status on serum concentration of proinflammatory cytokines in mice. A: Interleukin-6, B: Interleukin-1. Data are means ± SEM (Controls, Dominants and Subordinates: n=6). *p < 0.05: significant difference between dominant/subordinate animals and controls. ^#^ p < 0.05: significant difference between dominant and subordinate animals.

As displayed in Figure **5-A**, evaluation of IL-6 concentration in serum of experimental subjects showed that IL-6 levels in dominant (mean ± SEM: 275.75±40.07 pg/ml) and subordinate (mean ± SEM: 446.55±98.8 pg/ml) mice have increased significantly compared to control mice (mean ± SEM: 6.371±3.239 pg/ml; p<0.05); and this increase was observed significantly more in the subordinate group than the dominant group (p=0.05). In addition to the high levels of IL-1β concentration (Figure **5-B**) in subordinate (mean ± SEM: 55.275±17 pg/ml; p=0.01) and dominant (mean ± SEM: 22.75±5.053 pg/ml; p<0.05) mice compared to controls (mean ± SEM: 0.167±0.167 pg/ml), IL-1β concentration was more in serum of subordinate mice than dominant animals (p<0.05).

## Discussion and Conclusion

Animals as well as humans sense differences in social situations via a bio-psycho-neuro-social phenomenon [35-39]. In the current study, we investigated whether behavioral response to pain could be modulated in a model of dominant-subordinate relationships between pairs of conspecifics in a cage [15]. Additionally, we evaluated the health effect of this dominant and subordinate social status through measures of serum concentration of corticosterone, proinflammatory cytokines IL-6 and IL-1β. The behavioral analysis showed that subordinate Balb/c mice were hypoalgesic in the chronic phase of formalin test compared to control and dominant mice. On the other hand, dominant mice were hypoalgesic compared to subordinate mice during acute phase of formalin test (i.e., this is true only for the first time point assessed during this acute phase and the whole acute phase showed that subordinate mice are hyperalgesic). Both dominant and subordinate mice had elevations in corticosterone, IL-1 and IL-6 concentrations in serum compared to control group.

Body weight gain changes were investigated as chronic social stress is often associated with body weight loss [40,41]. This trend was confirmed in the present study. The subordinate mice displayed a significant decrease in body weight at the end of the stress period compared to control animals. The dominant mice also showed reduced body weight at the end of the stress experience but this decrease was not statistically significant. This finding is contradictory to the study by Savignac et al. in which body weight loss was more pronounced in animals with a dominant status [40]; such a discrepancy to the literature may be due to different social stress protocols and further investigations are needed in this regard. In general, our data suggest that dominant mice may develop a better coping strategy to deal with stress as compared to subordinates.

Despite the evidence that dominant and subordinate animals show different hormonal, physiological and behavioral profiles, data on whether these animals respond similarly to the same stressor are sparse and entangled [42-44]. Social stress in animal models is also known to induce increased serum corticosterone in chronically stressed animals regardless of the social status [40,45].In the present investigation, both dominant and subordinate subjects displayed a distinct increase in corticosterone levels compared to control group, confirming that both groups of animals were stressed by the chronic social interaction. This result suggests that while functional Hypothalamic-Pituitary-Adrenal (HPA) axis response to repeated stressor is an increased negative feedback leading to reduced corticosterone response [40], dominant and subordinate mice may have an impaired HPA axis response.

Similarly, the proinflammatory cytokines concentrations in serum showed an increase of both IL-6 and IL-1β levels in both subordinate and dominant mice compared to controls, with the subordinates displaying higher increases than dominants. It has been widely described that social stress can impair immune response in a status-dependent way, with subordinate mice being the most susceptible to immune alteration, an effect which can be explained by the immunosuppressive effect of increased corticosterone levels [40]. In this regard, social defeat has been shown to affect inflammatory immune processes, including variations of pro-inflammatory cytokines IL-1β, IL-6, and TNF-α in blood, lung, spleen, and brain [46]. IL‐1 and IL‐6 are the most important cytokines that can stimulate the HPA axis independently or synergistically, and these cytokines have autocrine effects in that they stimulate their own secretion from the cells that produce them. However, glucocorticoids inhibit the production of IL‐1 and IL‐6 by a negative feedback mechanism [47,48]. Social stressors may not only increase proinflammatory activity, but also alter the regulation of this response. Indeed, social stressors may interfere with the glucocorticoid-driven inhibitory process, and thereby lead to simultaneous elevations in glucocorticoid and proinflammatory activity. In other words, social stressors can decrease the ability of glucocorticoids to reign in proinflammatory response [47]. The increased IL-6 and IL-1β concentrations in both the subordinates and the dominant mice in the present study seem to confirm such findings.

Studying the effects of social stressors may be important to all social species, especially considering the robust social factors affecting pain sensitivity in humans, and in light of recent evidence suggesting the impact of social factors in rodent pain models [15,49,50]. The impact of stress on pain sensitivity is well established; stress has been observed to inhibit or exacerbate pain perception depending on the nature and/or parameters of the stressor. Indeed, it would be advantageous to inhibit pain behavior in a potentially dangerous situation in order to facilitate escape, whereas in other circumstances vigilance to painful stimuli might be more beneficial [51]. Formalin test as a model of chronic inflammatory pain in which hind paw injection of formalin is used to assess intense, short-lasting (minutes to tens of minutes) persistent pain. The distinction between visceral and peripheral pain, however, is important to the discussion for behavioral studies, in which an understanding of pain mechanisms is the ultimate goal [52]. During formalin test, peripheral activation of nociceptors, through a reduction in their transduction thresholds leads to hyperalgesia [53]. Moreover, stress is able to directly or indirectly activate visceral sensitive and/or nociceptive afferents inducing visceral hyperalgesia [54]; as it can initiate inflammatory changes or enhance the severity of pre-existing mucosal lesions. Alternatively, chronic stress affects peripheral pain perception differently in a model of formalin test [55]. To our knowledge, no previous studies have reported the effect of social status on the late chronic phase of formalin test in mice. This study, however, revealed status-mediated differences in the late phase of formalin test. The dominants’ response in the chronic phase of the formalin test (from minute 15 to 35) was similar to control group. Subordinate mice, however, were hypoalgesic in the chronic phase of formalin test compared to dominant and control subjects. Neurobiological mechanisms that are thought to be involved in chronic nociceptive experience have been shown to differ markedly from those activated by acute pain [15]. The findings of this study suggest that impaired negative feedback of the HPA axis in subordinate animals may be the explanation for the hypoalgesia observed in the chronic phase of their formalin test. The overexpression of endogenous opiate after stress exposure has been suggested to participate in the regulation of stress reactivity [56]. Social stressors like subordination attenuates sensivity of immune cells to glucocorticoids, as inflammation of peripheral tissue leads to increased synthesis and axonal transport of opioid receptors in dorsal-root ganglion neurons, thus resulting in their up-regulation. Under such conditions, the number of nociceptor endings increases, and the perineural barrier is disrupted, which facilitates the access of opioid agonists to their receptors [57], and all these effects lead to enhanced hypoalgesic efficacy at the level of peripheral receptors in inflammation [58]. Although this study did not evaluate endogenous opiates changes in response to such social stressors, and the exact mechanisms of hypoalgesia induced by social defeat stress are currently not clear, it may be reasonable to suppose that the effect of social defeat stress on pain behaviors imply the activation of descending pain modulatory system for prolonged stress by an increase of proinflammatory cytokines. 

Stress can also cause hyperalgesia depending on the type of stressor, as well as its intensity and duration [59]. Our results showed that chronic pain perception in dominant mice was much higher than controls during the last 25 minutes (from minute 36 to 60) of formalin test. This elevated pain response lasted about 54 minutes after the initiation of the chronic phase of the formalin test in dominant animals (data is not displayed in Figure **3**). Recent studies have led to a clearer understanding of possible mechanisms by which the host response to environmental and social stresses is mediated [60]. One modulator of stress and important variable in the link between the environment and individual body is serotonin. The effect of serotonin changes has been linked to aggressive behavior, chronic pain, and social dominance [61]. Pronociceptive or hyperalgesic effects of prolonged stress result in habituation or hyperactivity of the pain modulation system, in which serotonergic neurons in nucleus raphe magnus and endogenous opioid system may be modified [62]. It is well known that serotonin is a neurotransmitter for inhibitory neurons and is involved in the pain-modulation system [63]. Low blood serotonin levels are found in patients experiencing migraines and fibromyalgia syndrome. Brain serotonin is depleted in the area of the dorsal raphe nucleus, and it seems that this accounts for chronic pain [63]. Individuals that are more prone to aggression may be characterized by a serotonin deficiency [64], and it seems that the increase of chronic pain scores in formalin test of dominant mice is related to a decrease in serotonin levels during and after the confrontations.

In animals, repeated exposure to stressors like social subordination was shown to affect acute pain and produce hyperalgesia. While the mechanisms of stress-induced analgesia have been widely described, those underlying stress-induced hyperalgesia remain poorly understood [65]. Additionally, this study displayed some effects of social status on acute pain. We found that the experience of repeated defeat caused hyperalgesia in the subordinate subjects, which was different from dominant mice. Such results are contrary to Gioiosa et al. study that reported hypoalgesia in defeated intruders and hyperalgesia in dominants [15]. These differences may be explained by the type of pain evaluated: i.e., we scored pain behaviors whereas Gioiosa utilized the licking time during formalin test. Additionally, there were differences in the strains of mice and the dosage of formalin. Further investigations are needed to explanation of this disparity in results.

Taken together, the present study adds to the knowledge regarding the relationship between social subordination and pain, showing a negative association with chronic pain. Additionally, our data displayed a correlation with proinflammatory cytokines and an increase in corticosterone concentration. In addition to this type of behavioral and physiological modification in subordinates, there was an increase in chronic pain perception in dominant mice despite the fact that proinflammatory cytokines and corticosterone levels were increased. Therefore, further investigations are needed to understand elusive mechanisms of hypoalgesia and hyperalgesia after social stress.
